# Nanozymes with bioorthogonal reaction for intelligence nanorobots

**DOI:** 10.52601/bpr.2021.200044

**Published:** 2021-02-28

**Authors:** Si Sun, Xinzhu Chen, Jing Chen, Junying Wang, Xiao-dong Zhang

**Affiliations:** 1 Tianjin Key Laboratory of Low Dimensional Materials Physics and Preparing Technology, School of Sciences, Tianjin University, Tianjin 300350, China; 2 Tianjin Key Laboratory of Brain Science and Neural Engineering, Academy of Medical Engineering and Translational Medicine, Tianjin University, Tianjin 300072, China

**Keywords:** Nanozymes, Bioorthogonal reaction, Nanorobotics

## Abstract

Bioorthogonal reactions have attained great interest and achievements in various fields since its first appearance in 2003. Compared to traditional chemical reactions, bioorthogonal chemical reactions mediated by transition metals catalysts can occur under physiological conditions in the living system without interfering with or damaging other biochemical events happening simultaneously. The idea of using nanomachines to perform precise and specific tasks in living systems is regarded as the frontier in nanomedicine. Bioorthogonal chemical reactions and nanozymes have provided new potential and strategies for nanomachines used in biomedical fields such as drug release, imaging, and bioengineering. Nanomachines, also called as intelligence nanorobots, based on nanozymes with bioorthogonal reactions show better biocompatibility and water solubility in living systems and perform controlled and adjustable stimuli-triggered response regarding to different physiological environments. In this review, we review the definition and development of bioorthogonal chemical reactions and describe the basic principle of bioorthogonal nanozymes fabrication. We also review several controlled and adjustable stimuli-triggered intelligence nanorobots and their potential in therapeutic and engineered applications. Furthermore, we summarize the challenges in the use of intelligence nanorobots based on nanozymes with bioorthogonal chemical reactions and propose promising vision in smart nanodevices along this appealing avenue of research.

## INTRODUCTION

Bioorthogonal chemistry is described as the chemical reactions that can occur under physiological conditions in the living system without interfering with or damaging other biochemical events happening simultaneously (Bertozzi 2011; Grammel and Hang 2013; Sletten and Bertozzi 2009). Bioorthogonal chemistry has gained great interest since its first appearance in 2003 for its mechanisms complementary to traditional genetic-based methods on the basis of the Central Dogma (Bertozzi 2011; Ellen M 2011; Hang *et al*. 2003; Li and Chen 2016; Munoz and Heck 2014; Prescher and Bertozzi 2005). Bioorthogonal chemistry has several unique advantages such as wide applicability to almost all kinds of biomolecules, various versatility in probe molecules out of imagination and suitable scalability for diverse functional annotation in living cells (Ramil and Lin 2013; Rebelein and Ward 2018). For instance, fluorescent protein, as a traditional and powerful tool for labeling and visualization of intracellular proteins of interest (POI), has its own restrictions such as that macromolecular structure of fluorescent protein can form a large steric hindrance to affect the structure and function of POI and that the tool is just applies to the both ends (N terminal and C terminal) of proteins, resulting in failing to achieve the specific mark (Lin *et al*. 2008; Sletten and Bertozzi 2009). Bioorthogonal chemistry reactions have provide superior alternatives, especially in site-specific labeling (Sletten and Bertozzi 2009; Xu *et al*. 2020; Zhang *et al*. 2015; Zheng *et al*. 2015). The unnatural sugars with biological functionality called as bioorthogonal chemistry reactions have been developed to label and detect cell-surface glycans that cannot be visualized with genetic methods (Devaraj 2018; Laughlin *et al*. 2008; Li and Chen 2016; Prescher *et al*. 2004). Up to now, there are numerous reviews to describe and summarize the development of bioorthogonal chemistry reactions (Kim and Bertozzi 2015; Ramil and Lin 2013; Zheng *et al*. 2015).

Traditionally, bioorthogonal chemistry has largely been viewed as two-component "ligation" reaction under physiological conditions (Bertozzi 2011; Li and Chen 2016). Saxon *et al*. proposed the improved bioorthogonal chemistry to describe the biocompatible Staudinger ligation reaction between an azide and a modified triphenylphosphine (Ellen M 2011; Hang *et al*. 2003; Saxon and Bertozzi 2000). The reaction has been applied to the attachment of peptides *in* *vitro* and the mark of sugar on the cell surface with restrictions of slow reaction rate and side effects (Sletten and Bertozzi 2009). Since then, large amounts of bioorthogonal chemical reactions that meet requirements of the living systems have been developed, especially the Cu(I)-catalyzed azide alkyne cycloaddition (CuAAC) (Kenry and Liu 2019). To avoid the cytotoxicity of Cu(I) ions, scientists have made significant progress in study of strain promoted cycloaddition reaction, the so-called “copper free click chemistry”, which has increased the biocompatibility and the reaction rate (Ellen M 2011). The ligation reaction based on tetrazine compounds called inverse electron-demand Diels–Alder reaction (IEDDA) has the extremely fast reaction rate and superior biocompatibility (Carell and Vrabel 2016; Jiang and Wang 2013; Oliveira *et al*. 2017). Furthermore, scientists paid attention to the transition metals and additional conditions including ruthenium (Ru), palladium (Pd), iridium (Ir), iron (Fe), gold (Au) and UV other than copper to catalyze bioorthogonal ligation reactions in living systems (Rebelein and Ward 2018; Vong and Tanaka 2020). The deprotection reactions (bioorthogonal bond cleavage reactions) have been rapidly emerging. In recent years, nanozymes with bioorthogonal reactions have been employed in various fields, especially being thriving in the field of intelligence nanorobotics. Here, we review intelligence nanorobotics based on specific nanozymes with bioorthogonal reactions in biological fields and provide new ideas for research directions in the future.

## BASIC PRINCIPLE OF BIOORTHOGONAL NANOZYMES

Bioorthogonal chemistry, as chemical reactions occurring in the living systems, offers a strategy for labeling and visualizing the biomolecular, therapeutic cells, tumors and bacteria in the living systems (Kenry and Liu 2019; Prescher and Bertozzi 2005; Wang *et al*. 2019). Nanometric catalysts, also known as nanozymes here, are vital tools for bioorthogonal chemical reactions. Thus, nanozymes with bioorthogonal reactions are called as bioorthogonal nanozymes, also as nanozymes. Different kinds of bioorthogonal reactions have been gratifyingly realized (Boyce and Bertozzi 2011; Li *et al*. 2018; Prescher and Bertozzi 2005). Transition metal catalysts (TMCs) show excellent performance in catalyzing bioorthogonal reactions in biosystems that TMCs-mediated reactions in living systems can rapidly catalyze chemical transformations that cannot be realized by natural enzymes for specific applications (Clavadetscher *et al*. 2016; Kenry and Liu 2019; Oliveira *et al*. 2017; Prescher and Bertozzi 2005; Tonga *et al*. 2015; Wang *et al*. 2018, 2019; Weiss *et al*. 2014b). However, the direct application of TMCs-mediated reactions in living systems is still challenging due to the limitation of biocompatibility, poor water solubility, catalyst stability and rapid efflux from living systems (Tonga *et al*. 2015; Wang *et al*. 2019). Challenges like poor water solubility, unstable catalytic properties and so on can be improved with specially designed nanometric scaffold incorporated with TMCs (Tonga *et al*. 2015). Thus, nanozymes can be easily engineered for their location in the targeted tissues or turning their catalytic properties with various signals (Li and Chen 2016; Sasmal *et al*. 2013; Wang *et al*. 2019). Bioorthogonal nanozymes show tremendous potential in the development of therapeutics or diagnostics (Tonga *et al*. 2015).

Two strategies were developed to fabricate bioorthogonal nanozymes (Wang *et al*. 2019), one is to use nanomaterials as heterogeneous bioorthogonal nanocatalysts, and the other is to use nanometric scaffolds to encapsulate the molecular TMCs (Eda *et al*. 2019; Gupta *et al*. 2018; Zhang *et al*. 2020). The first one has been employed for Pd- and Au-mediated chemistries, and the latter one is more suitable to fabricate nanozymes using TMCs that require specific ligands to perform catalytic function (Volker and Meggers 2015; Wang *et al*. 2018).

Palladium can catalyze a rich list of chemical processes (Wang *et al*. 2019). Yusop *et al*. first demonstrated the use of metal nanozymes for intracellular catalysis in complex biological systems (Rebelein and Ward^ 2018^; Yusop *et al*. 2011). The biocompatibility of polystyrene microspheres entrapped palladium nanoparticles (PdNPs) was exploited, creating fluorescently-labeled Pd^0^-microspheres (Ji *et al*. 2019; Perez-Lopez *et al*. 2017). Wang *et al*. designed Pd nanoparticles-imbedded macroporous silica nanoparticles, making sense to prodrug activation (Wang *et al*. 2018). These catalysts were able to perform two types of reactions in biosystems: the deprotection of caged groups and C–C bond formation through the Suzuki-Miyaura reaction (Wang *et al*. 2019). For example, these Pd^0^-catalysts can cleave allyloxycarbonyl (alloc)-groups, leading to the uncaging of fluorescent rhodamine 110 (R110) (Chatterjee and Ward 2016; Rebelein and Ward 2018; Yusop *et al*. 2011). These Pd^0^-loaded microspheres also catalyze the Suzuki–Miyaura cross-coupling, leading to the accumulation of a rhodamine-fluorophore (Chatterjee and Ward 2016; Lang and Chin 2014; Rebelein and Ward 2018; Yusop *et al*. 2011). Moreover, Pro-protected 5-fluorouracil (Pro-5FU) and N-Poc-protected gemcitabine (N-Poc gemcitabine) were uncaged by PdNPs-ploystyrene to induce anti-proliferation on cancer cells (Li and Chen 2016; Rebelein and Ward 2018; Weiss *et al*. 2014a, b). Gold nanoparticles (AuNPs) were generated inside a polystyrene resin and performed the excellent catalytic in local release of R110 as well as the activation of anticancer prodrugs (Ji *et al*. 2019; Ngo *et al*. 2018; Perez-Lopez *et al*. 2017; Wang *et al*. 2019). This demonstrates that gold nanoparticles linked to polystyrene matrix have potential application in *in vivo* drug-release (Rebelein and Ward 2018).

The other is to encapsulate molecular TMCs in nanometric scaffolds (Jeschek *et al*. 2016). Cao-Milán *et al*. designed a family of 2-nm gold nanoparticles (AuNPs) encapsulating hydrophobic TMCs including Ru and Pd catalysts with specially designed ligands including hydrophobic segment, biocompatible segment and interacting unit (Cao-Milán *et al*. 2020). The interacting unit and hydrophobic TMCs can be specially designed to perform different functions (Cao-Milán *et al*. 2020; Tonga *et al*. 2015). For instance, the interacting groups with pH response can be used to target the acidic environment and those with thermoregulation can be used to regulate the activation process that responses to temperature (Cao-Milán *et al*. 2020). In general, nanometric scaffolds are nanoparticles, yet protein can be also used as nanometric scaffolds. Okamoto *et al*. conjugated ruthenium catalysts into biotin to combine with point mutations on streptavidin to optimize the catalytic effect, resulting upregulation of the synthetic gene circuit (Okamoto *et al*. 2018). Eda *et al*. chose albumin as the protein scaffold combined with ruthenium catalysts. It proved that Ru-bound nanozymes can accumulate into cancer cell lines and activate prodrugs of anticancer agent (Eda *et al*. 2019).

## INTELLIGENCE NANOROBOTS TRIGGERED BY DIFFERENT STIMULATION

**Figure 1:**
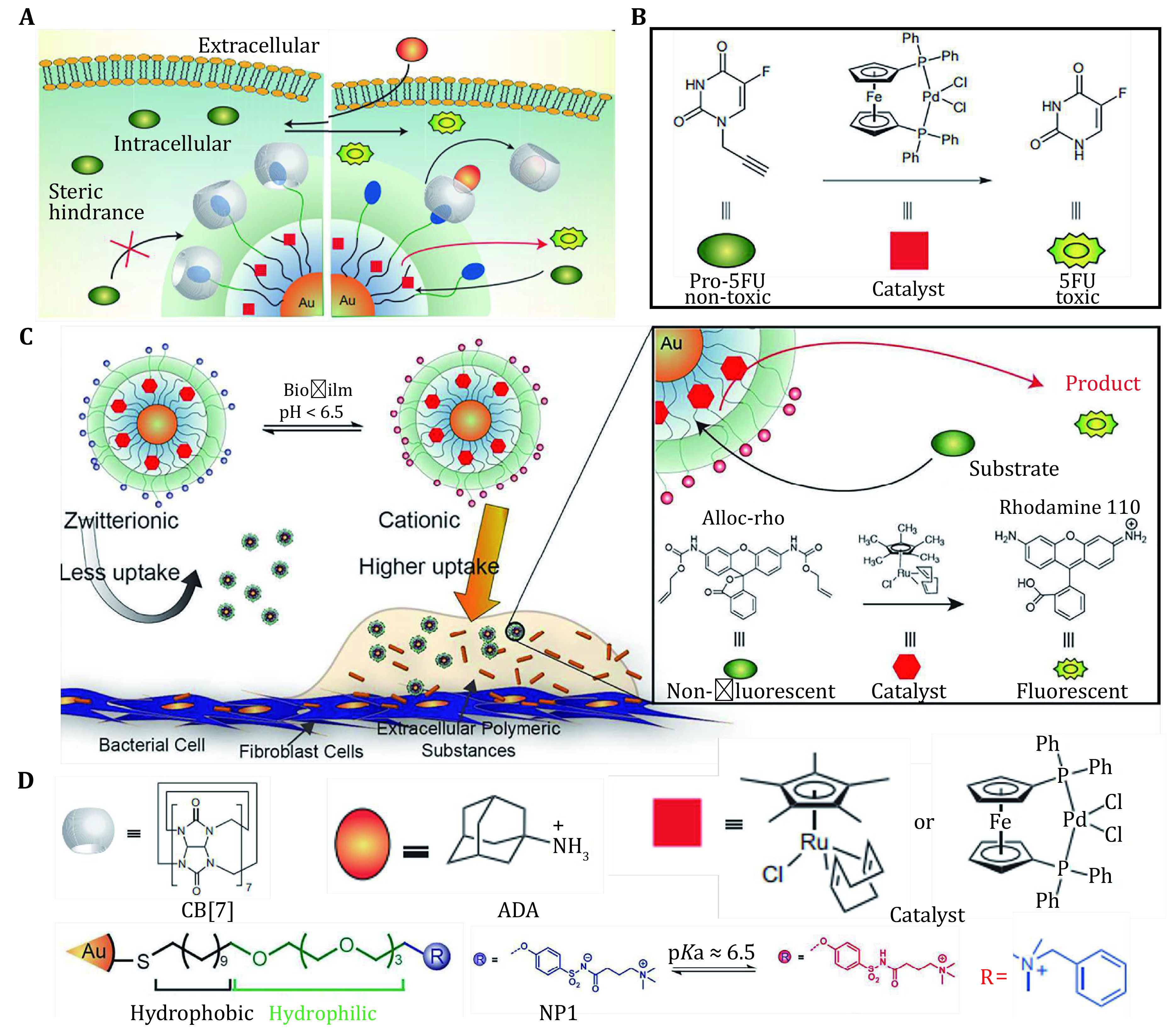
Chemical signals and pH-controlled nanorobotics. **A** Intracellular catalytic processes. Catalytic activity inhibited by steric hindrance of gatekeeper CB[7] and recovery of catalytic activity after adding ADA which can combine with CB[7], translating non-toxic prodrug into toxic drug. **B** Non-toxic prodrug activated by Pd catalyst to toxic drug. Reproduced with permission of Nature Publishing Group (Tonga *et al*. 2015). **C** Selective targeting of infected biofilms of Au nanorobotics mediated by change of the surface charge in different pH and translation of pro-fluorophores to fluorescent products identify their catalytic activity. Reproduced with permission of American Chemical Society (Gupta *et al*. 2018).) **D** Structure of CB[7], ADA catalyst (Ru and Pd), and the universal constituent parts of Au nanoreactor. Reproduced with permission of Nature Publishing Group (Tonga *et al*. 2015), and American Chemical Society (Gupta *et al*. 2018)

Nanorobotics sometimes is referred to as molecular robotics, an emerging technology field dealing with the design, simulation, control and others at or near the scale of a nanometer (Ghosh and Fischer 2009). The terms nanobot, nanomachince, nanowire or nanomite have also been used to describe such devices currently under research or development (Hoop *et al*. 2018; Soto *et al*. 2020; Yarin 2010). Intelligence nanorobots here are recognized as nanomachines that can perform controlled and adjustable stimuli-triggered response automatically regarding to different physiological environments or that can achieve some specific or special functions in physiological processes. Using nanomchines to perform precise tasks in the human body is seen as the frontier in nanomedicine (Unciti-Broceta 2015). Bioorthogonal catalysis provides new ways of mediating artificial transformations in living systems (Unciti-Broceta 2015). Combined with bioorthogonal reactions, intelligence nanorobots are endowed with more preponderance (Hoop *et al*. 2018). Nanozymes with the ability to regulate catalytic activity through chemical and physical signals can provide a biomimetic and dynamic control of bioorthogonal reactions (Wang *et al*. 2019). Wang *et al*. developed a first-in-class nanobot, a family of gold nanoparticles (AuNPs) encapsulating hydrophobic TMCs including Ru and Pd catalysts which can control their catalytic activity through a supramolecular host-guest strategy (Wang *et al*. 2019). Actually, the authors have employed the host-guest molecular recognition strategy to hide toxicity of gold nuclear with the help of CB[7] in 2010, the toxic AuNP-NH_2_ would be liberated when the competitive ADA was added to exert its effect. Combining with bioorthogonal reaction, the new devices were designed on basis of 2-nm AuNPs, featuring a hydrophobic alkane segment, a tetra　(ethylene glycol) unit and a dimethylbenzylammonium group (Fig. 1D), which are functionalized to bind with cucurit[7]uril (CB[7]) through host-guest chemistry as well (Tonga *et al*. 2015; Unciti-Broceta 2015). The complexation of nanozymes with CB[7] in this system blocks the access of substrates to the catalytic site, resulting in the complete inhibition of catalytic activity. However, this inhibition was reversible after the addition of 1-adamantylamine (ADA), a competing guest molecule, removing CB[7] away and allowing substrate access to catalytic sites (Fig. 1A) (Tonga *et al*. 2015; Wang *et al*. 2019). Using the catalyst, substrate was transformed into luminous or curative product, identifying the function of this strategy. This kind of nanorobot using supramolecular gated-activation system has been applied to living systems, such as intracellular prodrug activation in Hela cells (Fig. 1B) (Tonga *et al*. 2015). The release of catalyst could be intelligently controlled by addition of ADA, but the catalyst can’t be recycled after being freed. Asier Unciti-Broceta proposed that soft spring linkers could be used as confinement of catalyst (Unciti-Broceta 2015). When the host-guest system is applied into living bodies, selectivity and stability should be considered as well to avoid the side effect of drugs or catalysts.

Based on AuNPs encapsulating a ruthenium catalyst into the ligand monolayer, Gupta *et al*. developed pH-responsive sulfonamide-functionalized AuNPs through changing the modification of terminal groups on AuNPs surface (Gupta *et al*. 2018). The nanodevice was performed based on 2-nm AuNPs featuring terminal groups, which are functioned to selectively target the acidic microenvironment of biofilms (Tonga *et al*. 2015). Using the catalyst, targeting of the biofilm is achieved through charge-switchable NPs that transition from zwitterionic (nonadhesive) to cationic (adhesive) at the pH value typically found in biofilms and imaging studies of biofilms were performed on the basis of fluorophore (Rhodamine 110) through deallylation of a nonfluorescent precursor (Fig. 1C). The intelligent nanorobots based on bioorthogonal nanozymes provide an effective imaging system and enhance the fluorescence signal output (Gupta *et al*. 2018).

**Figure 2:**
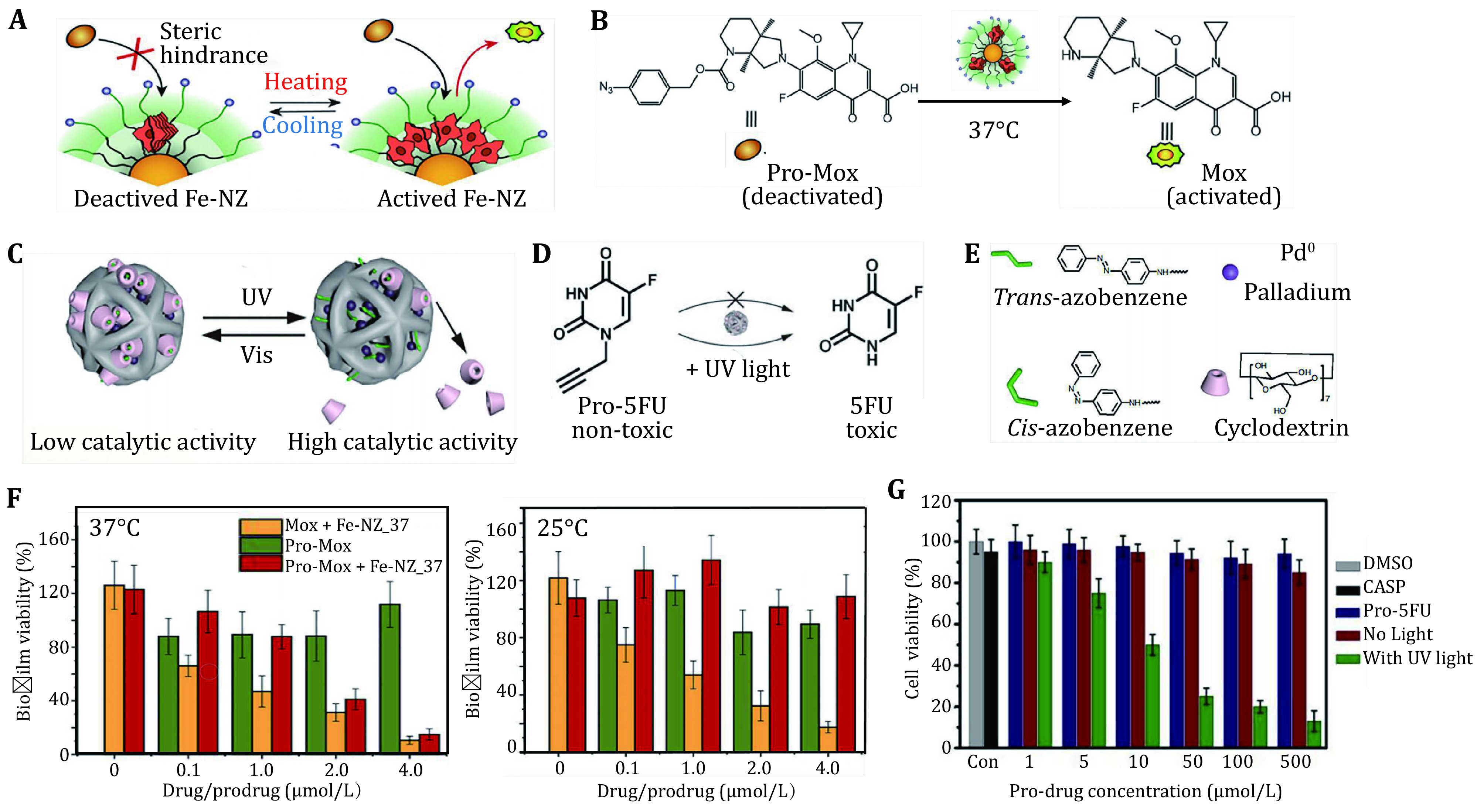
Temperature and light signals controlled nanorobotics. **A** The mechanism of substrate catalyzed by structurally variable nanorobotics under different temperature. **B** Catalyst presents activity toward prodrug in 37 °C. Reproduced with permission of Elsevier Inc. (Cao-Milán *et al*. 2020). **C** Conversion between *trans*-azobenzene and *cis*-azobenzene under UV light and visible light induces disaggregation and recombination of cyclodextrin which is used for conceal catalyst. **D** Prodrug activated by nanorobotic to toxic form under UV light. **E** Structure of trans-azobenzene, cis-azobenzene, palladium (Pd) and cyclodextrin (CD). Reproduced with permission of Nature Publishing Group (Wang *et al*. 2018). **F** Biofilm viability of different treatment groups under 37 °C and 25 °C. Reproduced with permission of Elsevier Inc. (Cao-Milán *et al*. 2020). **G** Cell viability with different treatment showing controlled catalytic activity. Reproduced with permission of American Chemical Society (Wang *et al*. 2019)

Besides a nanorobotic whose catalytic activity can be regulated by chemical and physical signals or pH, Cao-Milán *et al*. further developed a new kind of bioinspired nanorobot that can integrate catalysts into the thermoresponsive nanoparticles to be controlled through endogenous or exogenous thermal control (Canaparo *et al*. 2019; Cao-Milán *et al*. 2020; Unciti-Broceta 2015). Cao-Milán *et al*. found that supramolecular assemblies between catalysts can be regulated utilizing the confinement provided by the monolayer (Cao-Milán *et al*. 2020). Researchers prepared nanozymes by incorporating iron (III) tetraphenyl porphyrin (FeTPP) into AuTTMA scaffolds to form a system that can self-assemble into stacked aggregates through catalyzing the reduction of aryl azides (Cao-Milán *et al*. 2020). At the low temperature, FeTPP interacts with AuTTMA scaffolds, forming a compact structure to block the substrate access to the active site, thus deactivating the catalytic process (Cao-Milán *et al*. 2020). With the rising temperature, TMCs within the monolayer of nanoparticles are disassembled and redistributed, and thus the substrate has the access to the active site and the catalytic process are reactivated (Fig. 2A) (Cao-Milán *et al*. 2020). This kind of nanorobots using thermoresponsive gated-activation system with a resolution of 3 °C ranging from 25 to 27 °C has been applied to biological environments for antimicrobial uses (Fig. 2B and F) (Canaparo *et al*. 2019; Cao-Milán *et al*. 2020).

Wang *et al*. developed another nanorobot whose catalytic activity can be regulated by light-induced structural changes. They constructed the light-gated bioorthogonal catalysts by modifying microporous silica-Pd0 with supramolecular complex of an azobenzene (Azo) switch abbreviated as ASP and β-cyclodextrin (CD) (Fig. 2E) (Wang *et al*. 2018; Yan *et al*. 2020). Programmed nanozymes can be functionalized by CD through host-guest interactions and named as CASP (Wang *et al*. 2018). When the active site of CASP is occupied by CD, the catalytic activity will be inhibited. Under UV illumination, Azo can switch from *trans-* to *cis-*isomer and CD blocker will be released from the active site of CASP, leading to the reactivating of programmed nanozymes (Fig. 2C) (Wang *et al*. 2018, 2019). This kind of nanorobots using light gated-activation system has been successfully used for cell imaging and mitochondria-specific targeting agent by Suzuki–Miyaura cross-coupling reaction (Fig. 2D, G) (Wang *et al*. 2018, 2019).

## INTELLIGENCE NANOROBOTICS FOR BIOORTHOGONAL PRODRUG ACTIVATION

**Figure 3:**
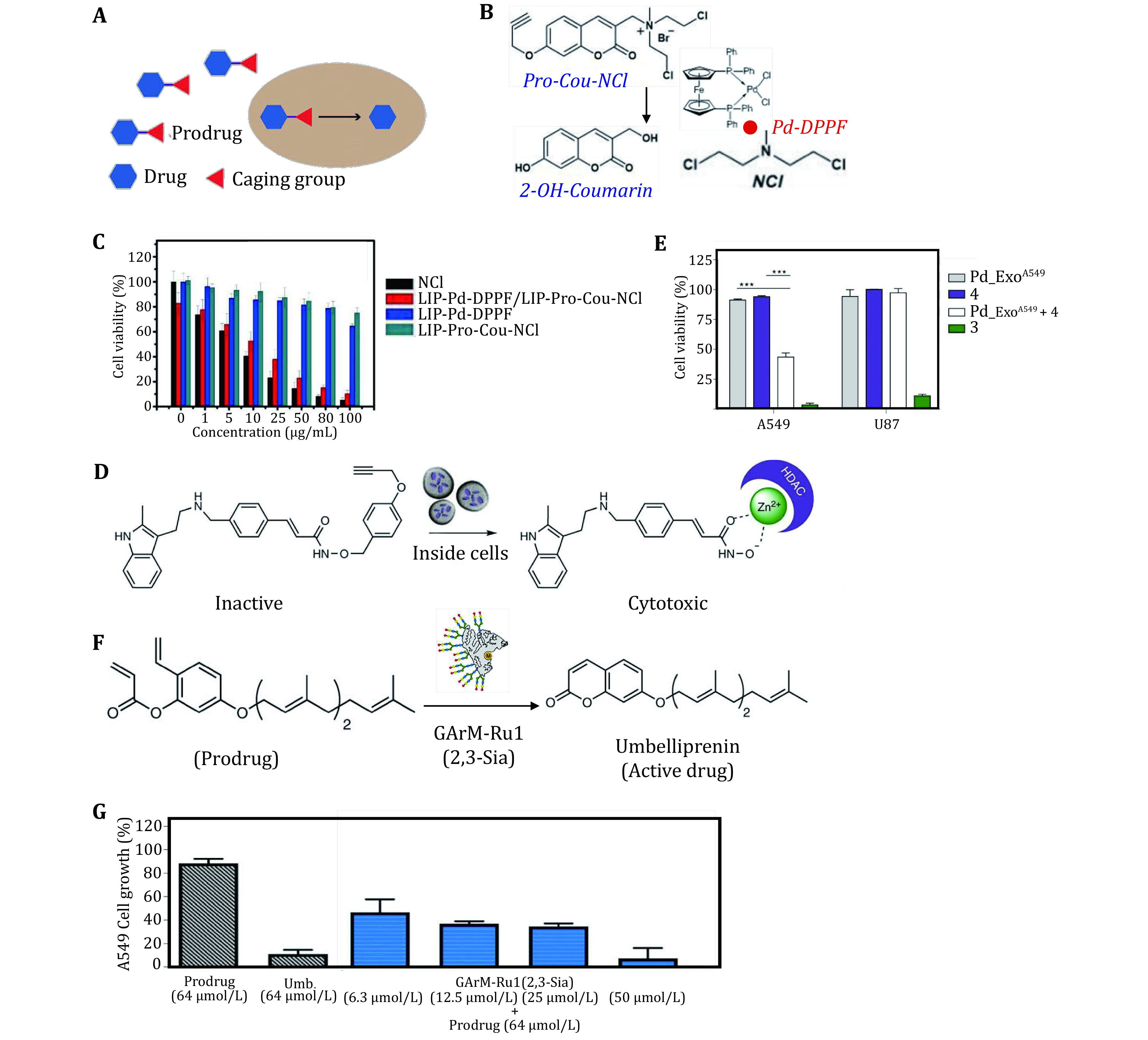
Bioorthogonal reaction used for prodrug activation. **A** Schematic diagram of prodrug activation. Reproduced with permission of Nature Publishing Group (Li and Chen 2016). **B** Reaction mediated by palladium catalyst and structures of pro-Cou-NCl, NCl and catalyst. **C** Intracellular toxicity introduced by the activated prodrug. Reproduced with permission of Elsevier Ltd. (Li *et al*. 2017). **D** Conversion of inactive prodrug to cytotoxic HDAC inhibitor under catalysis of Pd-Exo^A549^. **E** Selective action showed by incubating Pd-Exo^A549^ with cell A549 and U87. Reproduced with permission of Nature Publishing Group (Sancho-Albero *et al*. 2019). **F** Prodrug transformed to toxic umbelliprenin by ruthenium-bound GArM complexes. **G** Cellulo activity of A549 cells after different disposition. Reproduced with permission of Nature Publishing Group (Eda *et al*. 2019)

Prodrugs are defined as a class of compounds with no or low activity *in vitro* after modification, which are designed to prevent their parent drugs from decamping, reduce side effect and simultaneously improve biocompatibility and circulation half-life. Therefore, prodrugs are widely applied in the development of therapeutic drugs, especially in anti-tumor drugs (Bildstein *et al*. 2011; Li *et al*. 2017). After entering the body, prodrugs can be released through pH reaction, reduction reaction and other appropriate ways according to the difference between tumor microenvironment and normal tissue (Bildstein *et al*. 2011). Nevertheless, tumor heterogeneity inhibits further development of current strategies. The appearance of bioorthogonal reaction is significant to the development of new-type prodrugs for the help to construct chemical structure, relieve active groups through integrating exoteric stimuli to complementary bioorthogonal handles modification (Adam *et al*. 2018) and gather medicine at specific sites (Fig. 3A) (Das *et al*. 2019; Zheng *et al*. 2018). As described above, the transition metals as metalloenzymes attract great attention due to their unique catalytic properties, great biocompatibility and low toxicity in bioorthogonal cleavage reactions (Wang *et al*. 2015, 2019).

Li *et al*. constructed a bioorthogonal nanosystem for tumor imaging and inhibition by means of bond-cleavage reaction (Li *et al*. 2017). This system was comprised of two parts, Pd complex and prodrug, encapsulated by phospholipid liposomes separately facilitating internalization of drugs and catalyst and preventing bioorthogonal reaction from occuring prematurely. Pd-ferrocene was chosen as catalyst to deprotect a propargyl unit of prodrug, releasing a coumarin and an antineoplastic drug nitrogen mustard (NCl) which were applied as imaging agent to monitor the release of drugs and therapeutic drug to inhibit growth of tumor respectively (Fig. 3B). *In vitro* comparative experiments of different drugs showed that a large proportion of HeLa cells were killed under the combination treatment with prodrug and catalyst (Fig. 3C). Further study about the application of this system in mice found intratumoral injection of drugs could inhibit growth of tumor effectively. This process occurring inside tumor successfully couldn’t neglect the function of artificial positioning. The applications of Pd were also explored in other ways. Researchers have found that different kinds of exosomes can be produced by specific cells (Kalluri and LeBleu 2020), then the author anchored ultrathin palladium nanosheets inside the cancer-derived exosomes using the affinity between the donor cells and the exosomes. That way, exosomes can load metal into targeted cells without the disruption of physiological internal environment and endogenous products. The stability and reliability of hybridize vehicle were tested and it is found that panobinostat free was set through dealkylation reactions inside cells, and then the pharmacophore of the medicine emerged and chelated with zinc ion for its pharmacological action (Fig. 3D) (Sancho-Albero *et al*. 2019). Most importantly, it is proved that the prodrug can kill most of the A549 cells while it has little effect on U87 cells, exhibiting specific cell selectivity (Fig. 3E). Though the *in vivo* experiment was not carried out in this work, it is demonstrated that exosomes have enormous potential in the intelligent tumor treatments.

These two processes above are both achieved by bond cleavage reactions and have excellent outcomes. Some works based on the principle of bioorthogonal synthetic chemistry have wonderful functions as well. Eda *et al*. invented the GArM complexes with the help of ruthenium, which facilitated bioorthogonal ring-closing metathesis reaction and released umbelliprenin from its prodrug (Eda *et al*. 2019). The artificial metal nanozyme is composed of three parts: human serum albumin (HSA) providing protective pocket for catalyst, transition metal ruthenium acts as catalyst and complex N-glycans helping nanozyme target and accumulate in the specific cells (Fig. 3F). Incubated different kinds of cancer cells with the addition of prodrug and metal-based nanozymes, the anticarcinogen exerted good antineoplastic function and inhibited growth of cancer cells effectively (Fig. 3G).

Some scientists integrate the bioorthogonal reactions to the tumor microenvironment differences, creating peculiar drug release systems. Yao *et al*. reported a novel strategy of prodrug activation through employing the *trans*-cyclooctene (TCO) to cage Dox (Yao *et al*. 2018). The prodrug would be liberated by tetrazine (Tz) in an over-expressing endogenous phosphatase condition. Motivated by their work, some other schemes have also been proposed (Dong *et al*. 2020; Taran *et al*. 2019). The results are also of great significance but beyond the scope of this discussion, so that it will not be elaborated much here. In a word, the bioorthogonal reactions play an important role in designing diverse tactics to activate prodrugs *in vivo* and even *in situ*. In addition to studies mentioned above, some other metalloenzymes were employed to catalyst prodrugs with unique properties and various systems deserve to be pondered and further studied for the delighted future of their usage in clinic (Destito *et al*. 2019; Szponarski *et al*. 2018; Weiss *et al*. 2014b). Sufficient confidence should be placed in the development prospect of bioorthogonal chemistry in prodrugs activation.

## INTELLIGENCE NANOROBOTICS AND BIOENGINEERING

Bioengineering regarded as one of the most advanced technologies in the 21st century is a science-based discipline founded upon the biological sciences, including genetic engineering, protein engineering, cell engineering, and so on. Genetic engineering, protein engineering and cell engineering have been studied to combine with bioorthogonal chemistry and nanozymes, and have shown effective outcomes. Gene engineering, its extended concept-protein engineering and cell engineering are vital tools to introduce new products and functions that do not naturally exist, fabricate the structure of biomolecule and create novel processes to cure diseases (Nagamune 2017). Bioorthogonal reactions are famous for their relatively low influence on the normal biochemical reactions in living body. Therefore, some scientists manage to combine gene engineering with bioorthogonal reactions to compose intelligence nanorobotics.

**Figure 4:**
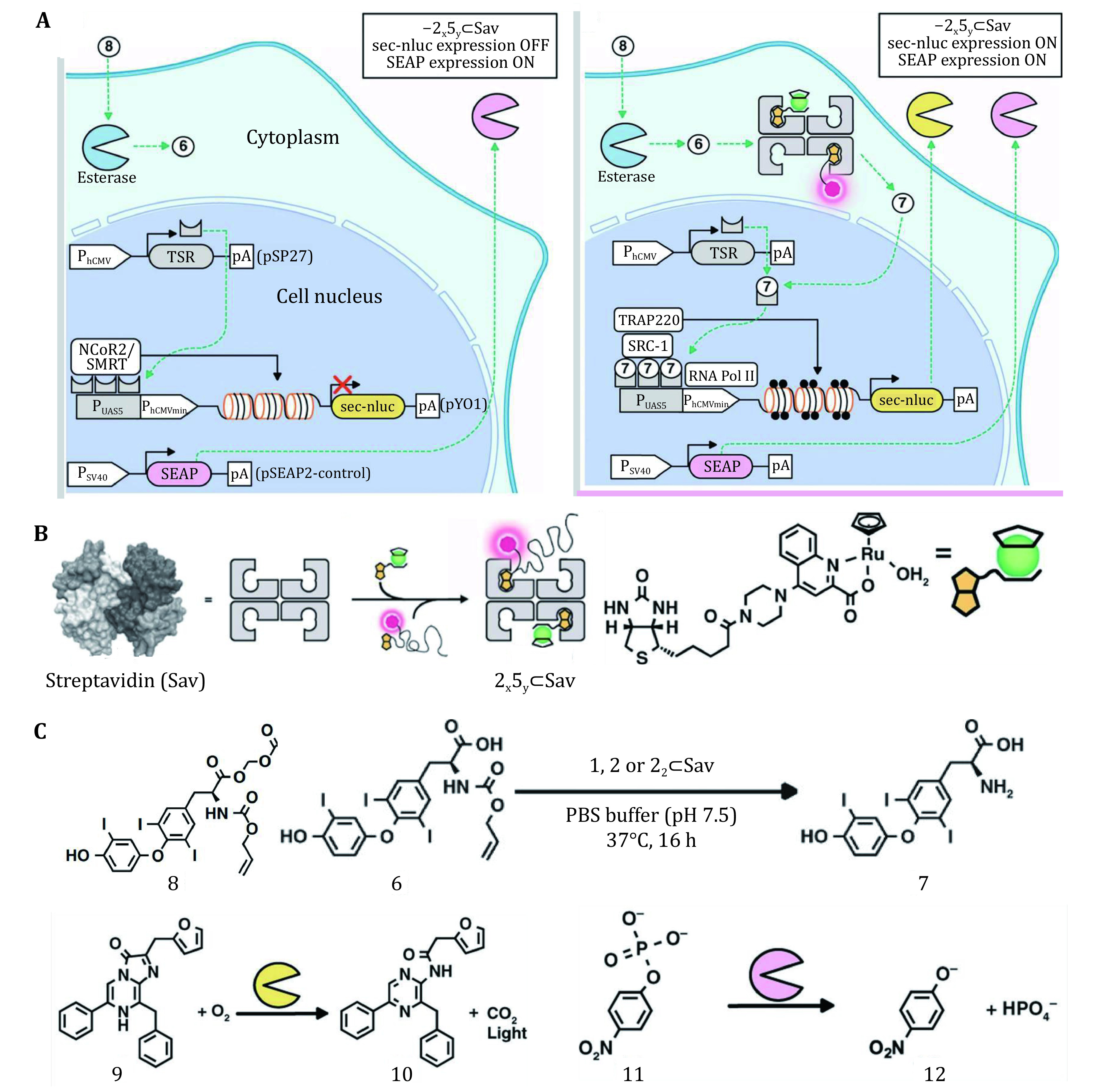
Bioorthogonal reaction used for genetic engineering. **A** Specific process of gene regulation catalyzed by Ruthenium complexes modified by biotin-streptavidin technology. **B** Structure of cell-penetrating biotin-streptavidin system and its two main components-Ruthenium complex and fluorescent TAMRA moiety. **C** Structure of compounds and relevant reactions used for constructing gene switch. Reproduced with permission of Nature Publishing Group (Okamoto *et al*. 2018)

It has been demonstrated that one kind of non-natural reaction-olefin metathesis can be catalyzed by artificial metalloenzymes designed based on principle of streptavidin–biotin, and the activity of the metalloenzymes could be regulated through directed evolution (Jeschek *et al*. 2016). However, this work only shows the application of metalloenzymes in the periplasm of *E. coli*. Two years later, creators of this method, Okamoto *et al*. constructed a cell-penetrating artificial metalloenzyme assisted by the biotin-streptavidin technology, integrating biotinylated ruthenium catalyst and biotinylated cell-penetrating TAMRA moiety to the structure of streptavidin, which participate in regulating a gene circuit in a designer mammalian cell (Fig. 4A) (Okamoto *et al*. 2018). The cell was transfected by a series of plasmids to fabricate a T3-gene switch, then detection of luminescence generated by biotinylated cell-penetrating moiety could verify if the metalloenzyme entered into designed cells. O-allyl carbamate cleavage mediated by nanozyme intracellularly produced product which mediated genetic circuit and relevant compounds were detected to verify if this gene switch was structured successfully (Fig. 4C). The results are extremely appalling because new functions might be constructed in the cell through this novel approach.

**Figure 5:**
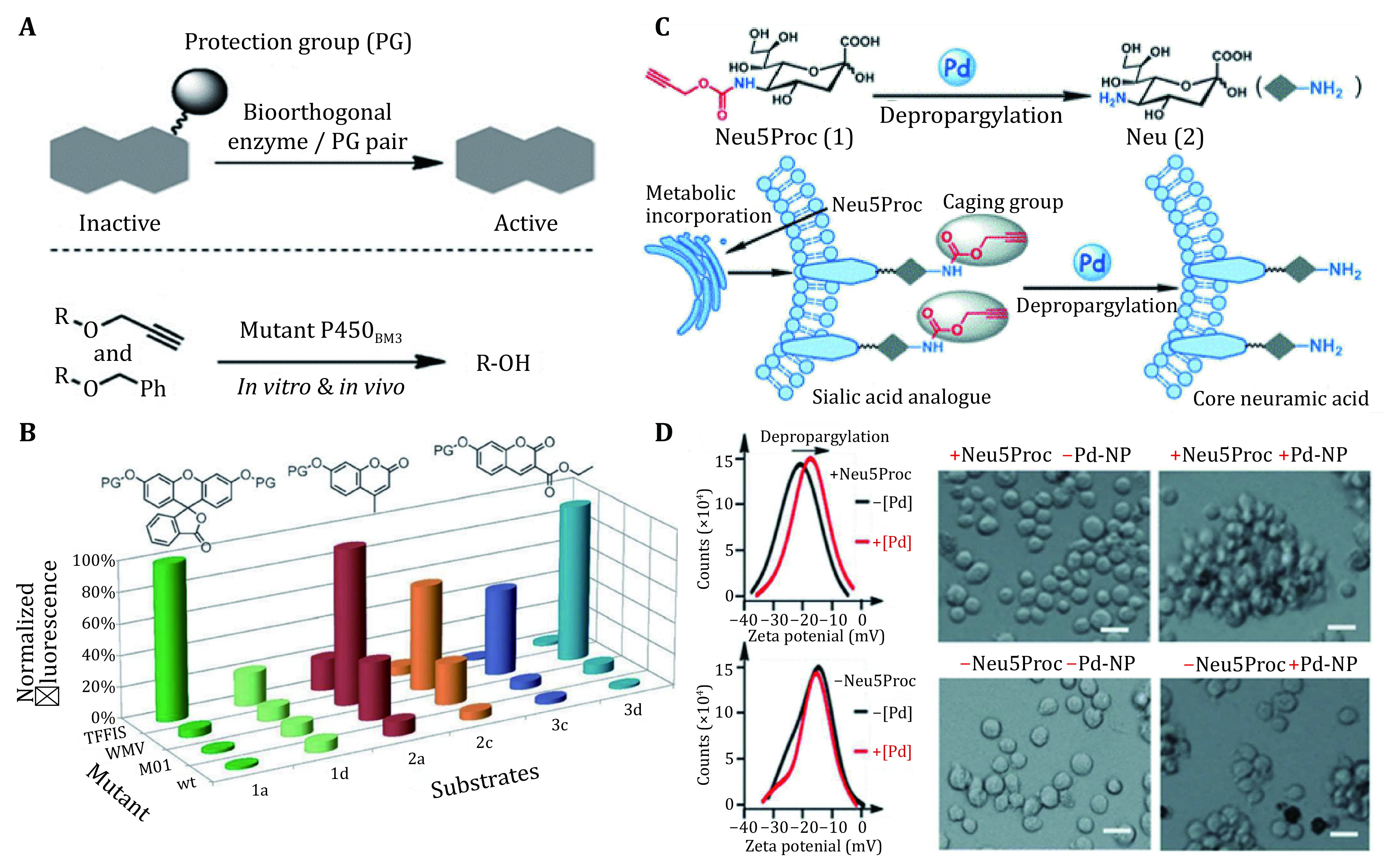
Bioorthogonal reaction used for protein engineering and cell engineering. **A** Basic principle of reaction mediated by bioorthogonal enzyme. **B** P450_BM3_ variants with various enzymatic activity designed with protein engineering catalyze substrates and produce different intensities of fluorescence. Reproduced with permission of John Wiley and Sons Ltd. (Ritter *et al*. 2015). **C** A chemical decaging strategy based on palladium-mediated reactions for generating Neu on live cells. Neu5Proc can be converted into the core neuramic acid and can be metabolically incorporated into cell-surface sialylated glycans. **D** Surface-charge variation and confocal microscopy imaging before and after treatment with Pd NPs. Pd-mediated depropargylation of surface-displayed Neu5Proc caused significant cell clustering that was not observed without Pd NPs or Neu5Proc. Scale bars: 10 μm. Reproduced with permission of John Wiley and Sons Ltd. (Wang *et al*. 2015)

In addition to combining with genetic engineering to modify the functions of organisms, protein engineering, closely related to genetic engineering has been employed as a strategy to enhance activity of the specific enzyme among bioorthogonal chemistry (Carrico *et al*. 2007; Wu *et al*. 2009). Ritter *et al*. attempted to utilize mutational CYPs to dismiss the protection group to expand the tool cabinet of the bioorthogonal reaction (Fig. 5A) (Ritter *et al*. 2015). Therefore, various variants were screened and then the activity toward different protective groups (propargylic and benzylic ethers) was tested to option the proper pairs which could be used in intracorporal or extracorporeal bioorthogonal reactions (Fig. 5B). Then several pairs of protective groups and enzymes were discovered, identifying that mutation of the bioorthogonal enzyme through protein engineering would promote their application range. Their work has obviously lightened the broad application prospect as a foundation to explore much more kinds of mutants and help to release uncaged structure by introducing a mutant into the body.

Recently, nanozymes with bioorthogonal reactions have been utilized to interact with cells (Wang *et al*. 2015, 2019). Nanorobots, in fact nanozymes with bioorthogonal reactions, can achieve the liberation of caged acids on cell-surface glycans (Wang *et al*. 2015). Pd-mediated depropargylation reaction can be used to generate neuramic acid (Neu) *in situ*, a unique type of sialic acid (Sias), on cell-surface glycans (Fig. 5C). N-Poc-neuraminic acid (Neu5Poc) could be metabolically incorporated into NeuN on cell-surface glycans as a mimic of N-acetylneuraminic acid (Neu5Ac). Upon the addition of a Pd catalyst, the Proc group can be cleaved from Nru5Poc to generate Neu *in situ*. Negative charges of carboxyl groups on Sias are particularly important for cell–cell repulsion among Sia-overexpressing cancer cells. The liberation of the amine in the negatively charged Sias could neutralize the negative charge and render the cells to clustering, and as expected, Jurkat cells incorporating Neu5Proc formed significantly clusters after Pd-mediated treatment (Fig. 5D). The intelligent nanorobotics provide a promising way for cell engineering.

Overall, the above have not been operated in practical experience, but they all show great potential for joint applications with biological nanobots in the near future. Apart from being used in prodrug activation and gene engineering, biorothogonal chemistry reaction practically has been studied in many ways: cell engineering (Szponarski *et al*. 2018; Wang *et al*. 2015, 2019), bio-labeling (Anhauser *et al*. 2019; Ma *et al*. 2017; Zhang *et al*. 2018), targeting specific sites (Chu *et al*. 2016) and so on. These have been discussed a lot by some other scientists (Kenry and Liu 2019; Li and Chen 2016), and will not be repeated here.

## FUTURE AND PROSPECTIVE

The concept of nanozymes was defined in 2013 as enzyme-like nanomaterials (Wang *et al*. 2019; Wu *et al*. 2019). In summary, bioorthogonal chemistry has rapidly developed to an unexcepted degree as a practical tool in the field of intelligence nanorobotics. Integrating bioorthogonal catalysts and chemical components into artificial nanostructures is a promising field and paves the way for the development of intelligence nanorobotics. To reach their potential, such man-made nanomachine needs to be specially designed with substrates. For example, light-controlled, pH-charged or thermally triggered nanorobots have been designed based on different mechanisms with various chemical molecules for applications in regulation of genetic engineering, activation of prodrugs and so on. Therefore, in this review, the history of bioorthogonal chemistry is recalled focused on ligation and cleave reactions and the paper involves nanozymes for fabrication and stimulated response and wide applications for intelligence nanorobotics in activation of prodrugs and regulation of engineering. Intelligence nanorobots might need specially a new and improved design to reach such stimulated-response control, such as photo-, light- or pH-responsive features or encapsulation two or more non-biological TMCs in one device (Unciti-Broceta 2015). Apart from automatic signal control, catalytic activity, potential toxicity, stability and solubility all need to be taken into consideration during the design of nanozymes for intelligence nanorobots. Biorthogonal chemistry help perform controlled chemistry in the conditions of biological functional groups and *in situ* within living organisms. Since its introduction, the toolkit of bioorthogonal reaction has been more practically useful and valuable. Broadly speaking, the fast-expanding bioorthogonal toolkit has promoted to think about multiple bioorthogonal reactions for various applications, particularly as kinetics, stability, structure and size that need to be further optimized. Moreover, targeting specific sites is a crucial and challengeable point for current research since only a few methods have been explored such as antibody-mediated reactions. In addition, considering the final purpose of creating intelligence nanorobots is to put into clinical use in living systems, it is important to highlight that their pharmacological behavior should be established under the condition of the design of nanozymes of intelligence nanorobots for chemotherapeutics (Wang *et al*. 2019). Pharmacological behavior includes: (1) the biodistribution of components of nanorobotics in organisms; (2) metabolic pathways and time of programed nanodevices in biosystems; (3) the action mode, effect and mechanism of programed nanorobotics in living systems; and (4) toxicology research in biosystems. Furthermore, catalytic activity and selectivity all need to be under consideration.

Programed nanodevices with bioorthogonal reaction for intelligence nanorobots is promising to make nanomedical devices out of non-biological components and perform tasks at specific locations in a precise manner (Unciti-Broceta 2015). However, one problem must be overcome before nanorobots can be applied into biomedicine is the ability of cell-targeting. Such features can be improved by introducing hydrophobic segments or molecules with specific targeting ligands, such as small molecules, peptides or DNA sequences. Despite these limitations, integrating biorthogonal catalysts and chemical compositions into artificial nanorobots are amazing. To sum up, transition metal catalysts served as biocompatible nanozymes provide opportunities to conduct abiotic catalysis in living systems and it is highly possible that smart devices with TMCs can modulate biochemical process with the ability to control catalytic properties upon changes in the environmental conditions without side effects in the future.

## Conflict of interest

Si Sun, Xinzhu Chen, Jing Chen, Junying Wang and Xiao-dong Zhang declare that they have no conflict of interest.
